# Protective immunity induced by DNA vaccine containing Tg*GRA35*, Tg*GRA42*, and Tg*GRA43* against *Toxoplasma gondii* infection in Kunming mice

**DOI:** 10.3389/fcimb.2023.1236130

**Published:** 2023-10-31

**Authors:** Youbo Shi, Jianbing Li, Weili Yang, Jia Chen

**Affiliations:** ^1^ The Affiliated People’s Hospital of Ningbo University, Ningbo, China; ^2^ Health Science Center, Ningbo University, Ningbo, Zhejiang, China

**Keywords:** *Toxoplasma gondii*, GRA35, GRA42, GRA43, DNA vaccine, protective immunity, Kunming mice

## Abstract

**Background:**

*Toxoplasma gondii* can cause congenital infection and abortion in humans and warm-blooded animals. *T. gondii* dense granule proteins, GRA35, GRA42, and GRA43, play a critical role in the establishment of chronic infection. However, their potential to induce protective immunity against *T. gondii* infection remains unexplored.

**Objective:**

This study aimed to test the efficacy of a DNA vaccine encompassing GRA35, GRA42, and GRA43 in inducing protective immunity against the highly virulent *T. gondii* RH strain (type I) and the brain cyst-forming PRU strain (type II).

**Methods:**

The eukaryotic plasmids pVAX-GRA35, pVAX-GRA42, and pVAX-GRA43 were constructed and formulated into two- or three-gene cocktail DNA vaccines. The indirect immunofluorescence assay (IFA) was used to analyze their expression and immunogenicity. Mice were immunized with a single-gene, two-genes, or multicomponent eukaryotic plasmid, intramuscularly. We assessed antibody levels, cytotoxic T-cell (CTL) responses, cytokines, and lymphocyte surface markers by using flow cytometry. Additionally, mouse survival and cyst numbers in the brain of mice challenged 1 to 2 months postvaccination were determined.

**Results:**

Specific humoral and cellular immune responses were elicited in mice immunized with single-, two-, or three-gene cocktail DNA vaccine, as indicated by significant increases in serum antibody concentrations of total IgG, IgG2a/IgG1 ratio, cytokine levels (IFN-γ, IL-2, IL-12, IL-4, and IL-10), lymphocyte proliferation, lymphocyte populations (CD4^+^ and CD8^+^ T lymphocytes), CTL activities, and survival, as well as decreased brain cysts, in comparison with control mice. Moreover, compared with pVAX-GRA35 + pVAX-GRA42, pVAX-GRA42 + pVAX-GRA43, or pVAX-GRA35 + pVAX-GRA43, multicomponent DNA vaccine with three genes (pVAX-GRA35 + pVAX-GRA42 + pVAX-GRA43) induced the higher humoral and cellular immune responses, including serum antibody concentrations, cytokine levels, lymphocyte proliferation, lymphocyte populations, CTL activities and survival, resulting in prolonged survival time and reduced brain cyst loads. Furthermore, mice immunized with pVAX-GRA35 + pVAX-GRA42, pVAX-GRA42 + pVAX-GRA43, or pVAX-GRA35 + pVAX-GRA43 showed greater Th1 immune responses and protective efficacy than the single-gene-vaccinated groups.

**Conclusion:**

These results demonstrate that Tg*GRA35*, Tg*GRA42*, or Tg*GRA43* are vaccine candidates against *T. gondii* infection, and the three-gene DNA vaccine cocktail conferred the strongest protection against *T. gondii* infection.

## Introduction

As an obligate intracellular parasite with worldwide distribution, *Toxoplasma gondii* can cause congenital infection and abortion in humans and warm-blooded animals (Elsheikha et al., 2021; Farhat and Hakimi, 2022). Most *T. gondii* infections in humans are asymptomatic, but infection of fetuses and immunocompromised people such as those with AIDS, organ transplantation, or neoplastic disease may cause severe disease or even lethal damage (Montoya and Liesenfeld, 2004; Dunay et al., 2018). Toxoplasmosis could also lead to considerable economic losses to the livestock industry (Tenter et al., 2000).

Chemotherapy is the mainstay of the control and prevention of toxoplasmosis. However, there are no available drugs that could eliminate *T. gondii* cysts in the infected host (Wang et al., 2019a). Therefore, vaccination is considered an effective strategy for the prevention and control of toxoplasmosis (Zhang et al., 2013; Zhang et al., 2015). There are not any commercial vaccines for humans; however, in sheep, there is one licensed vaccine based on attenuated-live *T. gondii* S48 strain, which shows considerable clinical efficacy (Buxton and Innes, 1995). Therefore, the development of an effective vaccine against *T. gondii* would be valuable for preventing infection in immunocompromised patients and reducing economic losses in food-producing animals. DNA vaccines have a good potential to prevent and control the parasite due to their ability to induce primarily Th1 cell-mediated immune and CD8^+^ cytotoxic T-cell (CTL) responses (Liu, 2011).

Dense granule proteins (GRAs) are found in the dense granules, which are cytoplasmic secretory organelles and play an important role in host cell invasion, virulence, and the formation of parasitophorous vacuoles (Hakimi et al., 2017; Farhat and Hakimi, 2022). Due to their key biological roles, several GRAs have been identified to be vaccine candidates for preventing toxoplasmosis in mouse models, including GRA15, GRA39, GRA24, and GRA25 (Chen et al., 2015; Xu et al., 2019; Zhu et al., 2021a). Three *T. gondii* dense granule proteins, GRA35, GRA42, and GRA43, are expressed in bradyzoites and tachyzoites and are individually required for induction of pyroptosis in Lewis rat macrophage and play a critical role in the establishment of chronic infection (Wang et al., 2019b). However, the assessment of their roles in protective immunity against *T. gondii* infection has not been performed.

Therefore, the objectives of the present study were to construct the eukaryotic plasmids pVAX-GRA35, pVAX-GRA42, and pVAX-GRA43 and analyze immune responses and protective efficacy in Kunming mice following DNA immunization with pVAX-GRA35, pVAX-GRA42, or pVAX-GRA43 against lethal challenge of *T. gondii* RH strain (type I) or chronic infection with *T. gondii* PRU strain (type II). In addition, two or three antigens were formulated as cocktail DNA vaccines, which were used to examine the protective efficacy against acute and chronic *T. gondii* infection.

## Materials and methods

### Mice

Specific-pathogen-free (SPF) female outbred Kunming mice, 6–8 weeks old, were purchased from Zhejiang Laboratory Animal Center, Hangzhou, China. All mice used for the experiments were handled humanely according to the Animal Ethics Procedures and Guidelines of the People’s Republic of China. This study was approved by the Animal Ethics Committee of Ningbo University (permission: SYXK(ZHE)2019-0005).

### Parasites, cells, and antigens

Tachyzoites of the *T. gondii* RH strain were revived from storage in liquid nitrogen and maintained in cell culture as previously described (Zhu et al., 2021a). Cysts of the *T. gondii* PRU strain were obtained from the brains of Kunming mice 1 month after oral inoculation with 10 cysts as previously described (Zhu et al., 2021b).

293-T cells were maintained in Dulbecco’s modified Eagle’s medium (DMEM; Invitrogen, Carlsbad, CA, USA) supplemented with 10% (vol/vol) heat-inactivated fetal calf serum (FCS), 100 IU/ml streptomycin, and 100 IU/ml penicillin at 37°C with 5% CO_2_.

For the preparation of *Toxoplasma* lysate antigen (TLA), tachyzoites of the RH strain were suspended in phosphate-buffered saline solution (PBS, 10 mM sodium phosphate containing 0.15 M NaCl, pH 7.2), sonicated (Sigma, St. Louis, MO, USA), and centrifuged at 2,100×*g* for 15 min, at 4°C. The supernatant containing TLA was sterile-filtered with 0.2 μm nitrocellulose filters (Sigma, St. Louis, MO,USA) and examined for protein concentration using an electronic spectrophotometer (Eppendorf, Hamburg, Germany) and kept at −80°C until further use.

### Construction of the eukaryotic expression plasmids

To construct the eukaryotic expression plasmids, pVAX-GRA35, pVAX-GRA42, and pVAX-GRA43, the full length of the coding sequence in the *T. gondii* GRA35 (Gene ID: TGGT1_226380), GRA42 (Gene ID: TGGT1_236780), and GRA43 (Gene ID: TGGT1_237015) gene were amplified by PCR from genomic DNA of *T. gondii* RH strain with three pairs of oligonucleotide primers (GRA35F, forward primer, 5′-CGGGGTACCATGGTCCTCAGTAACGGTTAC-3′; GRA35R reverse primer, 5′-TGCTCTAGAGGTTACCGTCAGTTACTAACTC-3′); (GRA42F, forward primer: 5′-GGGGTACCATGCGGATCCCTACTGTTA-3′; GRA42R, reverse primer: 5′-GCTCTAGAATCCGTTTGAGTACTCTC-3′); (GRA43F, forward primer: 5′-GGGGTACCATGAGGCTGAATCCCTTG-3′; GRA43R, reverse primer: 5′-GCTCTAGACTTATGGAGATTTCACTC-3′), and *Kpn*I and *Xba*I recognition sites were introduced and underlined. The PCR product was cloned into a pMD-18T vector (TaKaRa, China) and sequenced in both directions to ensure fidelity, generating pMD-GRA35, pMD-GRA42, and pMD-GRA43. These three fragments were cleaved by *Kpn*I/*Xba*I from pMD-GRA35, pMD-GRA42, and pMD-GRA43, respectively, and then subcloned into the *Kpn*I/*Xba*I sites of pVAX I (Invitrogen). After being transferred into *Escherichia coli* DH5α, these three plasmids pVAX-GRA35, pVAX-GRA42, and pVAX-GRA43 were processed by double restriction enzyme digestion to ensure positive clones, and then the obtained plasmids were purified by anion exchange chromatography (EndoFree Plasmid Giga Kit, Qiagen, Duesseldorf,Germany) following the manufacturer’s instructions, and were diluted with sterile PBS and stored at −20°C until use. The concentration of the eukaryotic expression plasmids was determined using a spectrophotometer at optical densities of 260 nm and 280 nm (OD_260_ and OD_280_).

### Expression of the eukaryotic plasmids *in vitro*

293-T cells were transfected with pVAX-GRA35, pVAX-GRA42, and pVAX-GRA43 or an empty vector (control plasmid, used as negative control) using Lipofectamine™ 2000 reagent (Invitrogen, Carlsbad, CA, USA) according to the manufacturer’s instructions as previously described (Chen et al., 2015). In brief, after 48 h posttransfection, cells were fixed with 100% acetone for 30 min and washed with PBS-0.1% Triton-X-100 (PBST) for three times and then were processed for indirect immunofluorescence assay (IFA) followed by incubation with goat anti-*T. gondii* tachyzoite polyclonal antiserum and a FITC-labeled donkey-anti-goat IgG antibody (Abcam, Cambridge, MA, USA). The specific fluorescence was examined using a Zeiss Axioplan fluorescence microscope (Carl Zeiss, Jena, Germany).

### DNA immunization and challenge infection

Mice were randomly divided into 10 groups of 35 mice each, and mice were immunized three times at 2-week intervals (at weeks 0, 2, and 4) with 100 μg eukaryotic expression plasmid DNA in 100 μl sterile PBS, 100 μg of the empty vector pVAX, or PBS (100 μl/each). One group of mice was not inoculated and used as a blank control. Blood was collected from the tail vein prior to each immunization and challenge infection, and sera were separated and stored at −20°C until analysis for specific antibodies.

As described previously (Zhu et al., 2021a), for each group of mice, 10 mice were challenged intraperitoneally (i.p.) with 1 × 10^3^ tachyzoites of virulent *T. gondii* RH strain, and the other 10 mice were challenged orally with 100 cysts of *T. gondii* PRU strain, and then the time of the death was recorded until a fatal outcome for all animals. The other six mice were challenged orally with 10 cysts of *T. gondii* PRU strain, 14 days after the last immunization, and the cysts in their brain were counted 30 days postchallenge.

Two weeks after the last immunization, a total of nine mice per group were sacrificed and splenocytes were aseptically harvested for flow cytometric and CTL activity analysis (three mice), lymphoproliferation assay (three mice), and cytokine measurements (another three mice).

### Measurement of humoral response

Serum samples were collected at four time points at 2-week intervals until the sixth week. Levels of IgG, IgG1, and IgG2a in serum samples from all immunized mice were determined using the SBA Clonotyping System-HRP Kit (Southern Biotech Co., Ltd, Birmingham, UK) as described previously (Zhu et al., 2021a; Zhu et al., 2021b). In brief, microtiter plates were coated with 100 μl (10 μg/mLl) solution of TLA diluted in PBS, overnight, at 4°C and blocked by PBS containing 1% BSA for 1 h at 37°C after washing three times with PBS containing 0.05% Tween20 (PBST). The plates were then incubated with the sera diluted by PBS for 1 h at room temperature, followed by washing three times by PBS again, and the anti-mouse-IgG, IgG1, and IgG2a horseradish peroxidase (HRP)-conjugated antibodies were added to each well for 1 h at 37°C. The binding was visualized by incubation with substrate solution (100 μl; pH 4.0; 1.5% ABTS, 1.05% citrate substrate buffer, 0.03% H_2_O_2_) for 30 min at room temperature. The absorbance was measured at 450 nm by using an ELISA reader (Bio-TekEL×800, USA). All samples were analyzed in triplicate.

### Lymphocyte proliferation assay

Two weeks after the last immunization, three mice per group were euthanized, and their splenocytes were aseptically harvested through a wire mesh and purified by removing the red blood cells using RBC erythrocyte lysis buffer (Sigma, USA). The lymphocytes from each group were then cultured in triplicate at a density of 2 × 10^5^ cells per well in a complete medium (DMEM medium 10% fetal bovine serum+ 100 IU/ml penicillin + 100 IU/ml streptomycin). The cells were stimulated with TLA (10 μg/ml), concanavalin A (ConA; positive control; 5 μg/ml; Sigma, St. Louis, MO, USA), or medium alone served as negative controls at 37°C for 72 h in a 5% CO_2_ incubator. After, 10 µl of 3-(4,5-dimethylthylthiazol-2-yl)-2,5-diphenyltetrazolium bromide (MTT, 5 mg/ml, Sigma, St. Louis, MO, USA) was added to each well and incubated for 4 h. The stimulation index (SI) was calculated using the formula (OD_570TLA_/OD_570Control_):(OD_570ConA_/OD_570Control_).

### Cytokine assays

Splenocytes were harvested as described for the lymphocyte proliferation assay, and different stimuli were added to the corresponding wells in flat-bottom 96-well microtiter plates (TLA, 10 mg/ml; medium alone for negative control). Culture supernatants were harvested, and the level of IFN-γ and IL-12 levels at 96 h, IL-2, and IL-4 levels at 24 h, and IL-10 was measured at 72 h as described previously (Zhu et al., 2021a; Zhu et al., 2021b). Cytokine concentrations were determined using commercial ELISA kits (Biolegend, San Diego, CA, USA) and reference to standard curves constructed with known amounts of mouse recombinant IFN-γ, IL-2, IL-4, IL-12, and IL-10. The analysis was performed with data from three independent experiments.

### CTL activity assays

After the preparation of spleen lymphocytes as mentioned above, the obtained lymphocytes were used for the measurements of CTL activity by using CytoTox96R Non-Radioactive Cytotoxicity Assay Kits (Promega, Madison, WI, USA), as previously described (Zheng et al., 2019). In brief, spleen cells were co-cultured with 100 U/ml recombinant murine IL-12 (eBioscience, San Diego, CA, USA), which were used as effector cells. Thereafter, Sp2/0 mouse cells transfected with eukaryotic expression plasmids were used as target cells (after 5 days) by using Lipofectamine™ 2000 reagent (Invitrogen, Carlsbad, CA, USA) according to the manufacturer’s instructions. After incubation of the mixture (containing effector cells and target cells at ratios of 10:1, 20:1, 40:1, and 80:1) for 6 h, the percentage of specific cell lysis was calculated using the formula (Experimental − Effector spontaneous − Target spontaneous)/(Target maximum − Target spontaneous) × 100.

### Flow cytometry

Flow cytometry was used to analyze the percentages of T-cell subsets CD4^+^ and CD8^+^ in the purified splenocytes obtained from mouse spleen in different groups as described previously (Zhu et al., 2021a; Zhu et al., 2021b). Splenocyte suspensions (5 × 10^5^ cells/ml) were stained with fluorochrome-labeled mAbs, including PE-CD3, APC-CD4, and FITC-CD8 (eBioscience, USA) for 30 min at 4°C in the dark. After washing with 2 ml PBS, the cultures were fixed with FACScan buffer (PBS containing 1% FBS and 0.1% sodium azide) and 2% paraformaldehyde. The samples were run in a FACScan flow cytometer (BD Biosciences, USA) and then analyzed for fluorescence profiles by SYSTEM II software (BD Biosciences, Franklin Lakes, NJ, USA). All samples were analyzed independently in triplicate, from three different mice.

### Statistical analysis

All statistical analyses were performed using Graph Pad Prism 5.0 and SPSS17.0 Data Editor (SPSS, Version X; IBM, Armonk, NY, USA). The differences in the data (e.g., antibody responses, lymphoproliferation assays, and cytokine production) between all the groups were compared by one-way ANOVA. Survival results are represented by Kaplan–Meier curves and were compared using the log-rank test. The results in comparisons between groups were considered different if *p* < 0.05.

## Results

### Identification of the expressed product by IFA

**Figure 1:**
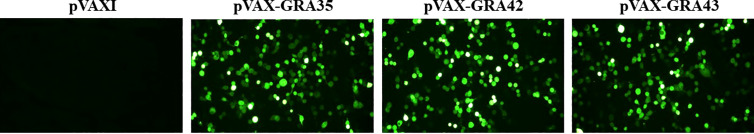
Detection of the recombinant Tg*GRA35*, Tg*GRA42*, and Tg*GRA43* proteins expressed in 293-T cells. 293-T cells were transfected with empty pVAX I, pVAX-GRA35, pVAX-GRA42, or pVAX-GRA43.

As shown in **Figure 1**, specific green fluorescence was observed in 293-T cells transfected with pVAX-GRA35, pVAX-GRA42, and pVAX-GRA43, whereas no fluorescence was observed in the negative controls transfected with the same amount of an empty pVAX I (**Figure 1**).

### Humoral response induced by DNA immunization

**Figure 2:**
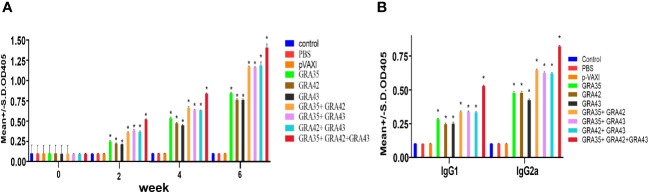
Detection of specific anti-T*. gondii* humoral immune responses induced by DNA immunization with single or multiple genes. **(A)** Determination of IgG antibodies in the sera of Kunming mice at 0, 2, 4, and 6 weeks. **(B)** Detection of IgG1 and IgG2a antibodies in immunized mice 2 weeks after the last immunization. ^*^ *p* < 0.05. Data are presented as the means ± SD.

To investigate humoral immune response in immunized mice, the levels of total IgG and subclasses IgG1 and IgG2a in all serum samples were determined by ELISA. As shown in **Figure 2A**, immunized mice showed a significantly higher serum level of specific IgG antibodies in comparison with the control groups (*p* < 0.05), with the highest IgG levels in the serum of mice vaccinated with three genes (pVAX-GRA35 + pVAX-GRA42 + pVAX-GRA43). Also, pVAX-GRA35 + pVAX-GRA42, pVAX-GRA35 + pVAX-GRA43, or pVAX-GRA42 + pVAX-GRA43 with significantly higher antibody levels induced by DNA immunization than with pVAX-GRA37, pVAX-GRA42, or pVAX-GRA43. Also, the increase in antibody levels occurred with successive DNA immunizations (*p* < 0.05). As shown in **Figure 2B**, the ratios of IgG2a/IgG1 in all immunized groups were higher, especially in pVAX-GRA35 + pVAX-GRA42 + pVAX-GRA43 group, compared with the control groups (*p* < 0.05).

### Cellular immune response

**Figure 3:**
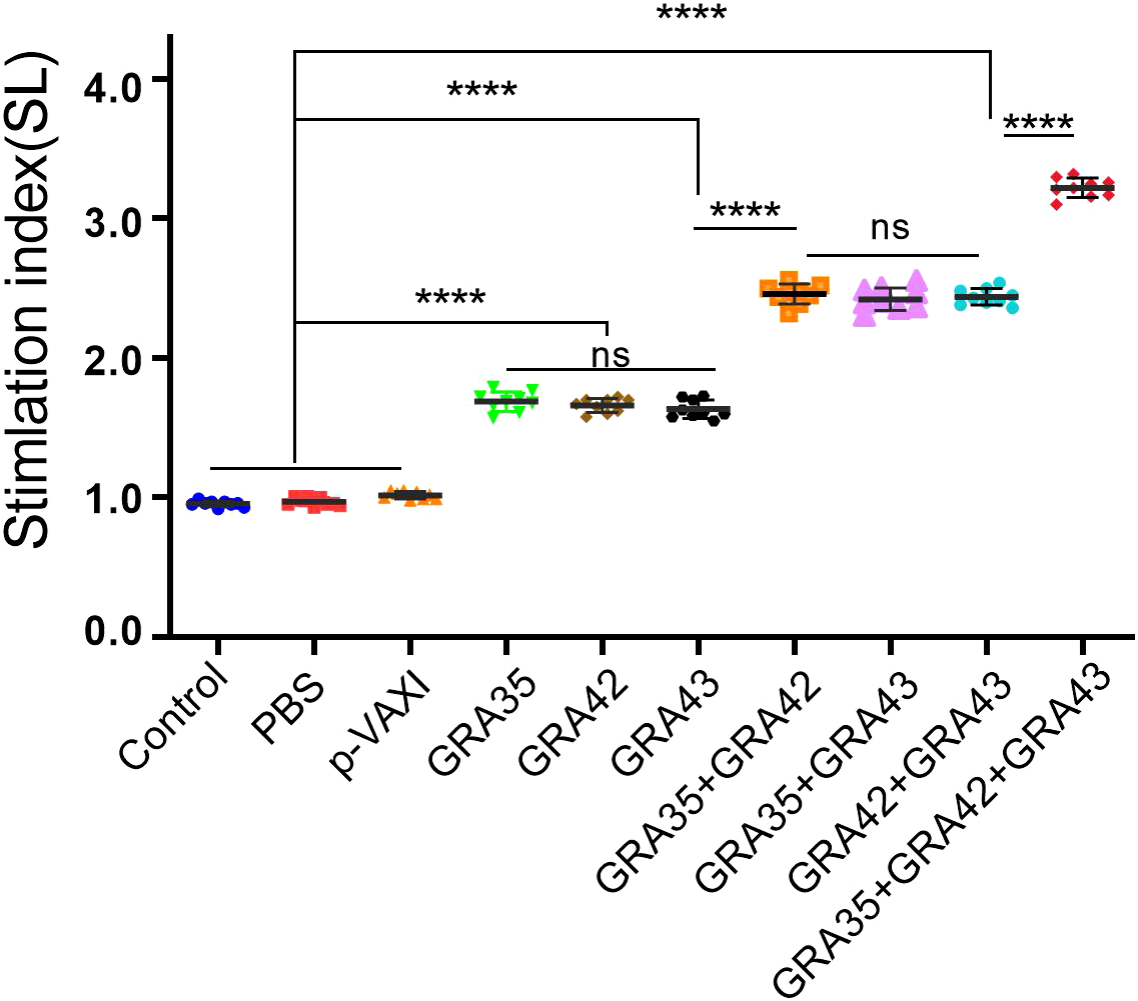
Splenocyte proliferative response in immunized and control mice. Lymphocyte proliferation stimulation index (SI). ****p < 0.0001. “n/s”, no significant. Data are presented as the means ± SD.

As shown in **Figure 3**, the SI in all two-gene immunized mice was higher than that in mice immunized with a single-gene plasmid or the control mice (*p* < 0.05). Also, the highest SI was induced in mice immunized with pVAX-GRA35 + pVAX-GRA42 + pVAX-GRA43. However, no significant difference in SI was observed between the pVAX-GRA35, pVAX-GRA42, and pVAX-GRA43 groups (*p* > 0.05). No significant difference in SI was observed between the three groups of two-gene immunized mice (*p* > 0.05).

**Figure 4:**
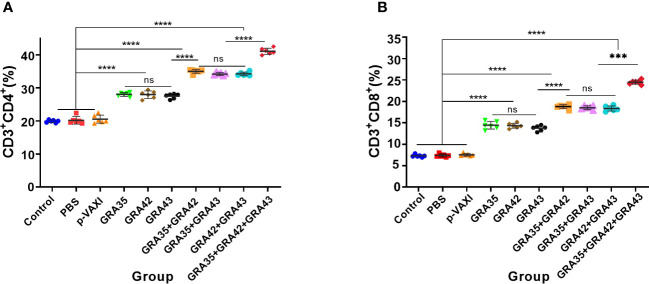
The percentages of CD4^+^ and CD8^+^ T cells in immunized and control mice. The percentages of CD4^+^ or CD8^+^ T cells in immunized **(A)** and control mice **(B)**. ***p < 0.001, ****p < 0.0001. “n/s”, no significant.. Data are presented as the means ± SD.

The percentages of CD4^+^ T and CD8^+^ T cells were significantly increased (*p* < 0.05) in all immunized mice in contrast to those in the control groups. DNA immunization with pVAX-GRA35 + pVAX-GRA42 + pVAX-GRA43 induced higher percentages of CD4^+^ T and CD8^+^ T cells than those in mice immunized with a two-gene plasmid or a single-gene plasmid (**Figures 4A, B**). However, there was no significant difference among the three control groups (*p* > 0.05).

### Detection of cytokine production and CTL activity

**Figure 5:**
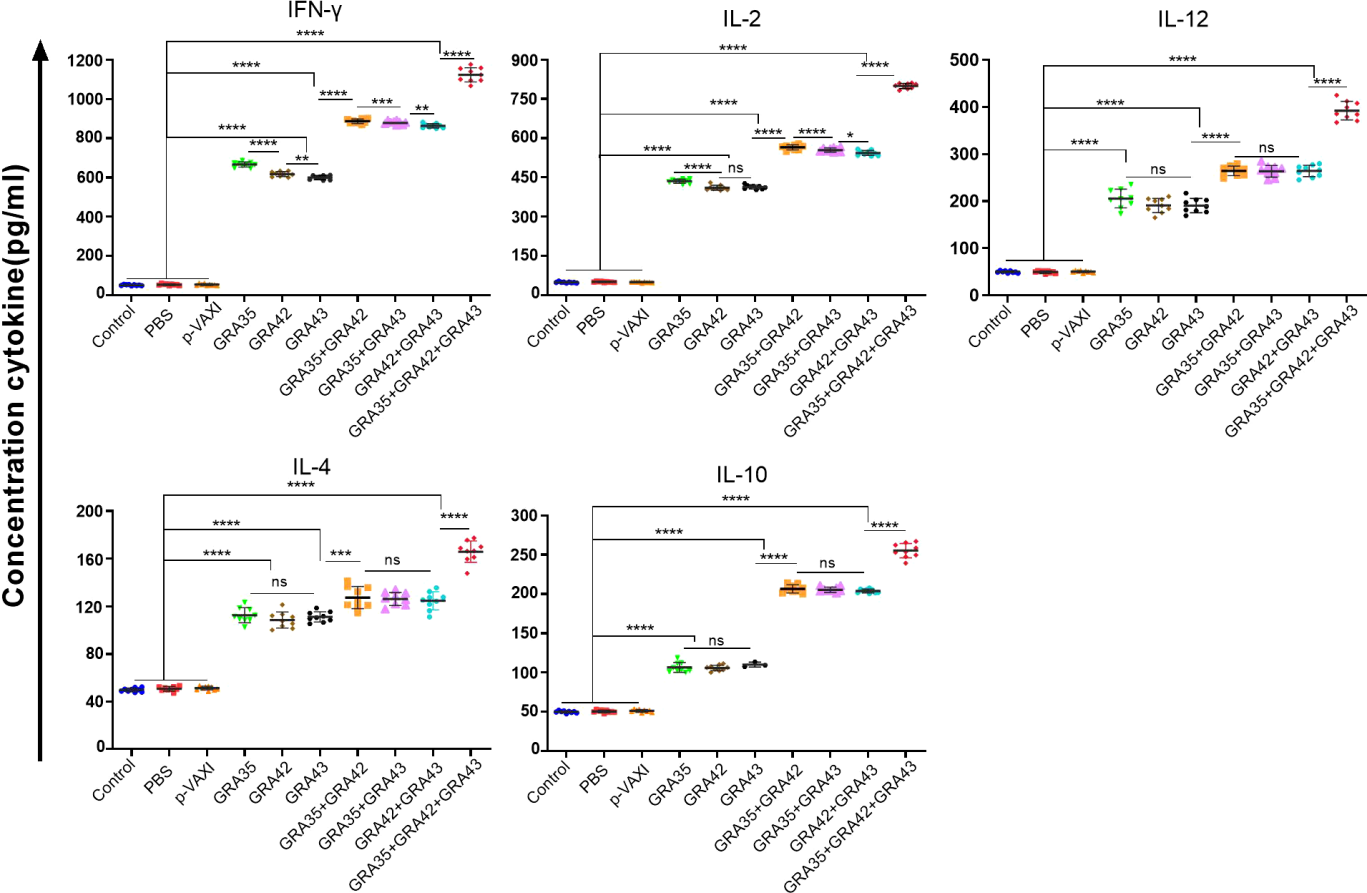
Cytokine production by splenocytes of mice immunized with single or multiple genes. *p < 0.05, **p < 0.01, ***p < 0.001, ****p < 0.0001. “n/s”, no significant. Data are presented as the means ± SD.

Splenocytes from immunized and nonimmunized mice were harvested 2 weeks after the last immunization for assessment of the levels of cytokines, including IFN-γ, IL-2, IL-12, IL-4, and IL-10. As shown in **Figure 5**, mice immunized with a three-gene cocktail of pVAX-GRA35 + pVAX-GRA42 + pVAX-GRA43 elicited the highest levels of IL-12, especially IFN-γ, and IL-2, followed by higher levels in two-gene immunized mice or single-gene immunized mice in comparison to those in the control groups (*p* < 0.05). In addition, Th2-type cytokine, IL-4, and a regulatory cytokine, IL-10, were also increased significantly in all immunized groups compared with the controls (*p* > 0.05). However, no significant differences were observed in the three control groups (*p* > 0.05).

**Figure 6:**
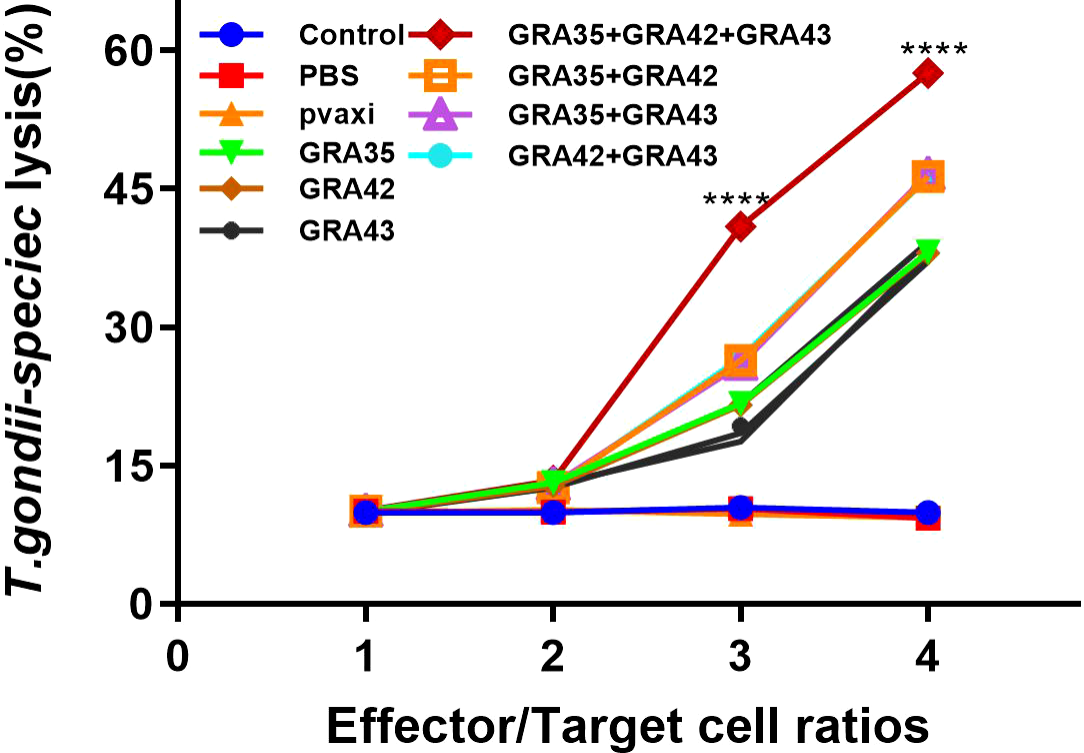
CTL activities of spleen lymphocytes in immunized mice. The effector-to-target cell ratios are indicated at the x-axis. The percentage of T. gondii- specific lysis is shown on the y-axis. ****p < 0.0001. Data are presented as the means ± SD.

The CTL activity of spleen cells in all immunized mice gradually increased in accordance with the gradual increase in the ratio of effector cells to target cells (the ratio at 80:1 matches the highest level of CTL activity). DNA immunization with pVAX-GRA35 + pVAX-GRA42 + pVAX-GRA43 induced higher CTL activity than that in mice immunized with a two- or a single-gene plasmid (**Figure 6**). However, there was no significant difference among the three control groups (*p* > 0.05).

### Protection of mice against challenge with *T. gondii* RH and PRU strain

**Figure 7:**
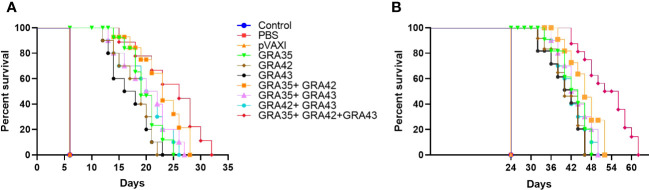
The survival rate of immunized Kunming mice challenged with 1 × 10^3^ tachyzoites of the RH strain **(A)** or 100 cysts of the PRU strain **(B)** 2 weeks after the final immunization.

After the i.p. challenge with 10^3^ tachyzoites of the virulent RH strain or 100 cysts of the PRU strain, the survival curve in immunized mice is shown in **Figure 7**. DNA immunization with eukaryotic expression plasmids significantly prolonged survival time compared with the control groups, which died within 6 days or 24 days postchallenge (*p* < 0.05). There was no significant difference among the three control groups (*p* > 0.05).

**Figure 8:**
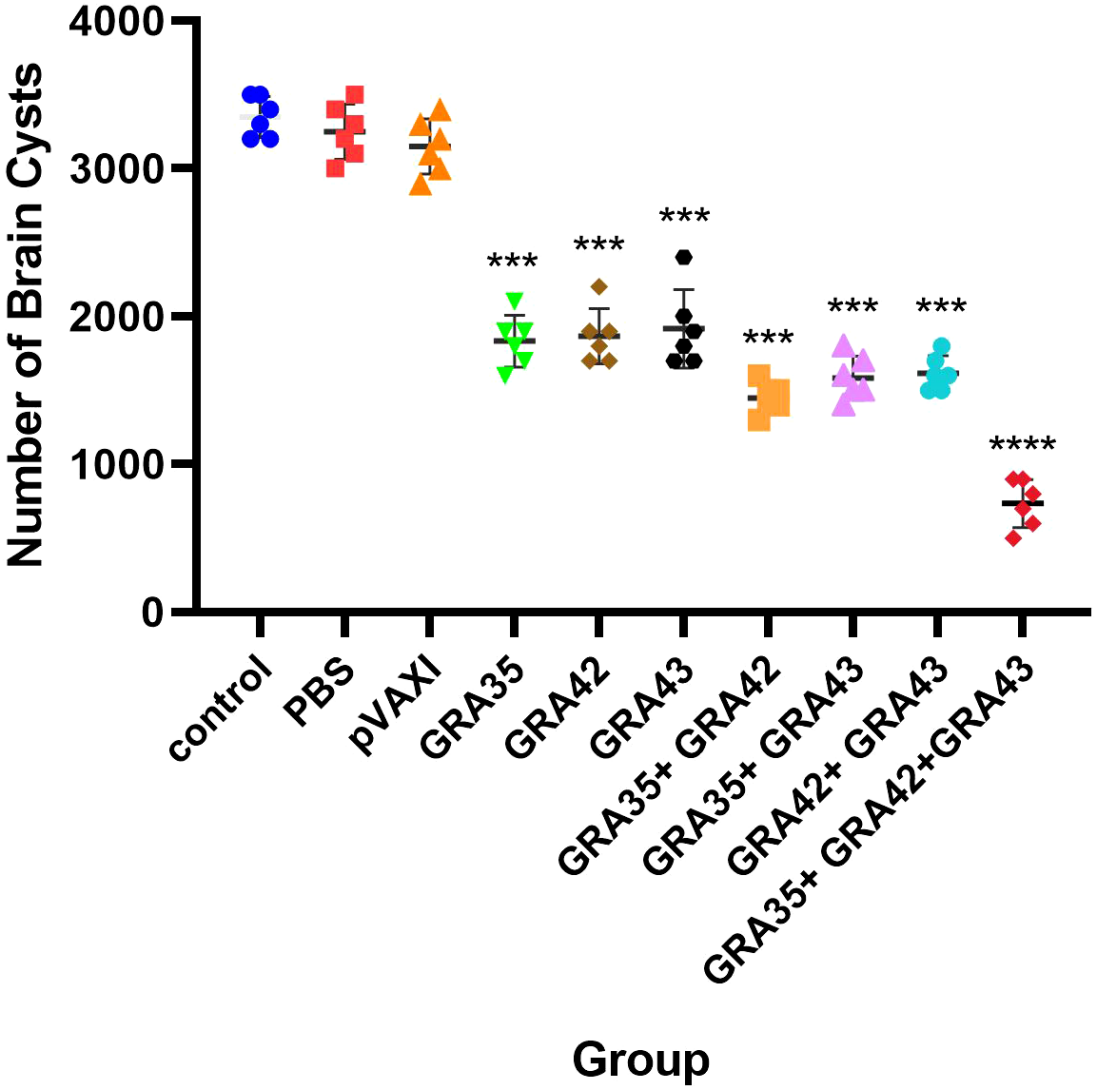
Protection against chronic toxoplasmosis in immunized mice 2 weeks after the final booster immunization. The bars represent the mean cyst burden per mouse brain after oral challenge with 10 cysts of the PRU strain. Cyst load was determined from whole brain homogenates of mice 4 weeks after the challenge. Data are means ± SD (representative of three experiments). ^***^ *p* < 0.001, ****p < 0.0001. “n/s”, no significant., compared with the control groups.

To evaluate the protective efficacy against chronic infection with the *T. gondii* PRU strain, tissue cyst loads were detected in the brains of experimental mice and controls 4 weeks after the third immunization. As shown in **Figure 8**, a significant reduction in the number of tissue cysts was observed in the brains of immunized mice with pVAX-GRA35 (41.8%), pVAX-GRA42 (40.7%), pVAX-GRA43 (39.2%), pVAX-GRA35 + pVAX-GRA42 (54.0%), pVAX-GRA35 + pVAX-GRA43 (51.2%), pVAX-GRA42+pVAX-GRA43 (48.7%), and pVAX-GRA35 + pVAX-GRA42+pVAX-GRA43 (76.7%), compared to the controls (*p* < 0.05). No significant difference was observed between the three control groups (*p* > 0.05).

## Discussion

Considerable research has been devoted to the development of DNA vaccines against *T. gondii* infection, in which i.p. challenge with *T. gondii* tachyzoites was often used in vaccination experiments, which does not mimic the natural infection route (Wang et al., 2019a). Therefore, oral challenge can mimic natural infection by ingestion of parasite cysts or cat-derived mature oocysts (Wang et al., 2019a). Also, the challenge dose of *T. gondii* plays a critical role in the analysis of the immune protective effect (Li and Zhou, 2018), and intragastrical inoculation of 80–100 cysts of PRU strain per mouse is often used to determine the survival in mouse model due to the low-virulence strain PRU. In the present study, we immunized Kunming mice with pVAX-GRA35, pVAX-GRA42, and pVAX-GRA43, or their two- or three-gene pooled vaccine, and estimated the protective efficacies against chronic toxoplasmosis in an established mouse model challenged orally with 10 or 100 *T. gondii* Pru tissue cysts. These mouse models infected with *T. gondii* have provided the benefit of observing a longer survival time than that in mice infected with a high dosage of the lethal *T. gondii* RH strain and also mimicking the natural route of *T. gondii* infection.

DNA and recombinant protein vaccine strategies have been widely used in past years (Li and Zhou, 2018; Zhang et al., 2023a) due to the low cost and effective induction of immune response. Although several antigens had been demonstrated as candidate vaccines against *T. gondii*, such as SAG4 (Zhou and Wang, 2017), DOC2C (Zhang et al., 2018a), ROP21 (Zhang et al., 2018b), ribosomal P2 protein (Yu et al., 2022), and GRA24 (Zheng et al., 2019), no studies have focused on the evaluation of the immune response and protective efficacy of Tg*GRA35*, Tg*GRA42*, and Tg*GRA43*. Therefore, we constructed the pVAX-GRA35, pVAX-GRA42, and pVAX-GRA43 plasmid, and IFA analysis using FITC-labeled secondary antibodies was used to detect their expression in cells (Tg*GRA35*, Tg*GRA42*, and Tg*GRA43*), suggesting that these constructed plasmids are able to successfully express the proteins *in vitro* and that these recombinant proteins have good immunogenicity and can evoke immune responses. It is well known that T-cell-mediated adaptive immune responses are critical to protect against *T. gondii* infection (Silva et al., 2009; Jongert et al., 2010). In this study, mice immunized with pVAX-GRA35, pVAX-GRA42, or pVAX-GRA43 induced a significant *T. gondii*-specific splenocyte proliferation, demonstrating that an adaptive immune response against *T. gondii* was elicited, which may contribute to effective cell-mediated immunity against *T. gondii* infection. Moreover, CTLs play a vital role in the control of infection with intracellular pathogen (Elsheikha et al., 2021). In particular, *T. gondii*-specific CD8+ CTL activity has been considered to play a critical role in controlling *T. gondii* replication and clearance (Jongert et al., 2010). Therefore, the induction of *T. gondii*-specific CTL is a significant strategy for the development of effective *T. gondii* vaccines. In our study, CTL activity was examined, and spleen lymphocytes prepared from the immunized mice showed higher CTL activity than the three controls, indicating that these DNA vaccine candidates induced a pathogen-specific CTL response and an effective immune response to intracellular pathogens. These results are similar to some previous studies in a novel mRNA vaccine with TGGT1_216200 mRNA-LNP and DNA vaccine with GRA24-based gene (Zheng et al., 2019; Zhang et al., 2023b).

Additionally, flow cytometry analysis of the proportion of CD4^+^ T cells and CD8^+^ T cells in immunized mice revealed significantly higher proportions in immunized mice than in the controls. Our findings have demonstrated that these activated cellular responses could provide protective immunity against *T. gondii* by NK cells and CD4^+^ T cells during acute infection, or by IFN-γ producing cells from CD8^+^ T cells and, to a lesser extent, CD4^+^ T cells in chronically infected mice (Gigley et al., 2009; Dupont et al., 2012). Moreover, the B-cell response is important in the anti-*T. gondii* infection response (Sayles et al., 2000). Also, the IgG antibodies against *T. gondii* infection via inhibiting attachment of the parasite to the host cell receptors or activation of the complement protein and opsonizing parasites by phagocytosis (Sayles et al., 2000; Zhang et al., 2018b; Zhu et al., 2021b). Furthermore, specific antibodies can play an essential role in cell-mediated immunity. In the present study, our immunized animals exhibited increased anti-*T. gondii* IgG levels in contrast to those of controls. Our further results on subclasses of IgG, where IgG2a was predominant over IgG1, have indicated that these DNA vaccine candidates could induce humoral immunoreaction mediated by Th1, which was considered to exert an essential role in host immune protection against *T. gondii* infection (Wang et al., 2019a; Zheng et al., 2019; Zhu et al., 2021a).

Generally, the activated T helper type 1 (Th1) cells are the host immunity effectors against intracellular bacteria and protozoa, including *T. gondii*, involving in Th1 type cytokines, IL-12, IL-2, and IFN-γ in response to antigen (Taylor et al., 2004; MacMicking, 2012; Sturge et al., 2013). IFN-γ is more critical for protective immunity than cytotoxicity-based effector functions both during the acute and chronic phases of *T. gondii* infection (Yap and Sher, 1999; Mishima et al., 2002; Tan et al., 2011). IL-12 can play a critical role in eliciting the production of IFN-γ by CD8+ T cells during acute *T. gondii* infection (Wilson et al., 2008).

IL-2 is also necessary for the development of memory T cells, which depends upon the expansion of the number and function of antigen-selected T-cell clones (Beadling and Smith, 2002). T helper type 2 (Th2) cells are distinct lineages of CD4^+^ effector T cells, with the secretion of IL-4, IL-5, IL-9, IL-10, IL-13, and IL-17E/IL-25, which are required for humoral immunity and play an important role in coordinating the immune response to large extracellular pathogens (Bessieres et al., 1997). During the acute phase of toxoplasmosis, the concomitant IL-10 and IL-4 responses dampen systemic type-1 cytokine production and prevent lethal immunopathology (Dupont et al., 2012). Therefore, our results of the production of IFN-γ, IL-2, IL-12, IL-10, and IL-4 in immunized mice suggest that the appropriate Th1 type and Th2 type cellular responses confer host resistance against *T. gondii* infection.

Theoretically, a multi-component vaccine containing more CTL epitopes may elicit better protective immunity against parasite infection than a single antigen due to the enhanced numbers of *T. gondii*-specific CTLs, thereby leading to elevated production of the antigen-specific cytokine IFN-γ (Jongert et al., 2010; Wang et al., 2019a). In this study, we have examined the protective efficacy of three or two DNA multicomponent vaccines against *T. gondii* infection, which boosted humoral and cell-mediated immune responses, resulting in a substantially decreased parasite cyst load in the brains and prolonged survival time of immunized mice in comparison with single gene-immunized infected mice, further showing that a cocktail DNA vaccine is a promising approach for control of *T. gondii* infection when compared to single-gene-based vaccination (Zhu et al., 2021b). In comparison with DNA vaccines expressing *T. gondii* GRA39 (Zhu et al., 2021a), or expressing *T. gondii* MIC5 and MIC16 (Zhu et al., 2021b), the three-gene cocktail DNA vaccine achieved a 76.7% reduction in the parasite cyst burden in our study. Furthermore, the reduction in the brain cyst load in this three-gene cocktail DNA vaccine was greater than the 55.3% reduction achieved by DNA vaccination with three genes, including *T. gondii GRA24*, *GRA25*, and *MIC6* (Xu et al., 2019), and even a 57.8% reduction in the vaccine candidate by using multiple antigenic peptides encapsulated by chitosan microspheres (Guo et al., 2018). Also, the three-gene-based DNA multicomponent vaccine showed the highest protective immunity, even though no significant differences were observed in two-gene-based DNA multicomponent vaccines, indicating that these elements are significant in the development of an effective vaccine.

## Conclusion

The recombinant plasmids encoding *T. gondii GRA35*, *GRA42*, and *GRA43* have significant potential to elicit humoral and cellular immunity against acute and chronic toxoplasmosis, suggesting that the immune efficacy of these DNA vaccine candidates should be further evaluated in other apicomplexan parasites. Also, further studies focusing on inducing a stronger protective immunity by co-administration with genetic adjuvant, for IL-33, IL-21\IL-15, and IL-7\IL-15 are needed.

## Data availability statement

The raw data supporting the conclusions of this article will be made available by the authors, without undue reservation.

## Ethics statement

The animal study was approved by The Animal Ethics Committee of Ningbo University (permission: SYXK(ZHE)2019-0005). The study was conducted in accordance with the local legislation and institutional requirements.

## Author contributions

YS and JC performed the experiments, analyzed the data, and wrote the manuscript. WY and JL took part in the acquisition of the data. YS and JC designed and supervised this research. All authors contributed to the article and approved the submitted version.
